# Sensor Screening Methodology for Virtually Sensing Transmission Input Loads of a Wind Turbine Using Machine Learning Techniques and Drivetrain Simulations

**DOI:** 10.3390/s22103659

**Published:** 2022-05-11

**Authors:** Baher Azzam, Ralf Schelenz, Georg Jacobs

**Affiliations:** Center for Wind Power Drives, RWTH Aachen University, 52074 Aachen, Germany; ralf.schelenz@cwd.rwth-aachen.de (R.S.); georg.jacobs@cwd.rwth-aachen.de (G.J.)

**Keywords:** machine learning, artificial intelligence, multivariate data analysis, feature importance, virtual sensing, drivetrain simulation, time-series simulation

## Abstract

The ongoing trend of building larger wind turbines (WT) to reach greater economies of scale is contributing to the reduction in cost of wind energy, as well as the increase in WT drivetrain input loads into uncharted territories. The resulting intensification of the load situation within the WT gearbox motivates the need to monitor WT transmission input loads. However, due to the high costs of direct measurement solutions, more economical solutions, such as virtual sensing of transmission input loads using stationary sensors mounted on the gearbox housing or other drivetrain locations, are of interest. As the number, type, and location of sensors needed for a virtual sensing solutions can vary considerably in cost, in this investigation, we aimed to identify optimal sensor locations for virtually sensing WT 6-degree of freedom (6-DOF) transmission input loads. Random forest (RF) models were designed and applied to a dataset containing simulated operational data of a Vestas V52 WT multibody simulation model undergoing simulated wind fields. The dataset contained the 6-DOF transmission input loads and signals from potential sensor locations covering deformations, misalignments, and rotational speeds at various drivetrain locations. The RF models were used to identify the sensor locations with the highest impact on accuracy of virtual load sensing following a known statistical test in order to prioritize and reduce the number of needed input signals. The performance of the models was assessed before and after reducing the number of input signals required. By allowing for a screening of sensors prior to real-world tests, the results demonstrate the high promise of the proposed method for optimizing the cost of future virtual WT transmission load sensors.

## 1. Introduction

Several recent trends in the field of wind energy motivate the need for monitoring the load situation within the WT drivetrain during operation. The increase in WT-rated power and rotor diameter over time has been a prevalent trend in the industry in recent decades and has resulted in an increase in the loads experienced by WT drivetrain components [1,2,3]. In addition, space limitations for wind farms have resulted in the commissioning of more densely packed wind farms [4]. Due to turbine interactions, such as wake effects, a high location dependency for the load situations experienced by WTs in different areas of a given wind farm arises [4,5]. Particularly of interest is the load situation within the main gearbox (GB) of the WT, as overloading can cause long downtimes, as well as high repair costs in the case of its failure, as compared to other WT components [6,7]. Whereas currently deployed WT condition monitoring systems aim to detect early signs of failure, they are not capable of measuring the 6-DOF GB input loads during operation, mainly due to the high cost of available direct measurement solutions [8].

On the other hand, solutions that aim to indirectly or virtually sense transmission input loads face a different challenge of collecting sufficiently representative data during WT operation to develop and validate their solutions [8]. Azzam et al. addressed this need by providing a methodology for generating the necessary data for virtual load sensor development using multibody simulations (MBS) of a Vestas V52 WT model subjected to simulated wind fields according to IEC 61400-1 [9]. The authors demonstrated that MBS simulations can be used to significantly reduce the resources needed during development of WT virtual load sensors by providing a more flexible, less costly alternative to physical measurement campaigns as a data source for virtual sensor prototyping. In this paper, we build on this finding by providing an approach for screening candidate sensors needed for virtually sensing 6-DOF WT transmission input loads prior to real-world testing. As the location of a sensor is an important feature of its design, candidate sensors refer not only to the type of measurement, e.g., distance or rotational speed measurement, but also to the location of the envisaged sensor, e.g., left or right torque arm of the gearbox. By identifying the most influential candidate sensors with respect to the accuracy of the virtual load estimator, in this investigation, we aimed to reduce the number of sensors required and hence the field deployment costs of virtual sensor systems for WT drivetrain load monitoring.

Machine learning has been demonstrated to be a highly relevant tool for data analysis in a variety of domains where data are abundantly available, such as finance [10], astronomy [11], and speech and character recognition [12,13,14]. More recently, machine learning, with the help of synthetic data generation tools, has also been successfully applied in various domains. For example, several computer vision investigations were able to achieve tasks such as object detection [15], robot grasping [16,17], and assembly [18] using synthetic data. In a recent highly publicized example, synthetic data in the form of fake impersonation videos, also known as deepfakes, was able to deceive not only humans but also facial recognition software [19]. In the previously discussed applications, the generation of synthetic data was primarily motivated by scarcity of the desired data or, in the case of deepfakes, the non-existence of the desired data. In this investigation, a virtual load sensor is envisaged for indirectly measuring WT transmission input loads via a set of practical and cost-effective sensors aimed at capturing the deformations and misalignments resulting from the targeted input loads during operation. The input variable selection or, in this case, the design of this set of sensors is crucial for the performance of the envisaged virtual sensor system [20]. Once a sensor set is designed, the resource-intensive instrumentation and testing phases can commence in order to collect empirical measurements that can be used to develop and test the virtual sensor algorithms. As a source of data, real measurements provide more realistic data than what can be obtained from a simulation model. However, physically testing competing combinations of candidate sensors is challenging [20].

Two main obstacles hinder the possibility of iteration through competing sensor sets using real-world testing. Although theoretically possible, changing the designed sensor set during testing is practically difficult due to reduced accessibility, as well as the high cost and limited nature of testing time, would need to be reduced to allow for reinstrumentation. In addition, a sufficient variety of operating conditions should be covered during testing, as changes in process characteristics and operating conditions were highlighted in a 2009 survey of engineers to be a major challenge during development and maintenance of virtual sensors [20]. This further extends the requirements and, hence, the costs of the physical tests for the envisaged WT transmission virtual load sensor. It also further motivates the need to screen candidate sensors prior to the costly instrumentation and real-world testing phases. Although domain knowledge is useful for selecting sensors or input variables for virtual sensing algorithms, it is difficult to understand the relationship between every input and output variable; therefore, a systematic methodology is needed for screening candidate sensors [20]. In this investigation, we address this need by presenting a methodology for sensor screening during the data-scarce virtual sensor design phase prior to real-world testing. Using the methodology recently published by Azzam et al. [9], simulated wind fields covering a variety of operating conditions were applied to an MBS WT model to generate a time series of displacements, misalignments, and other parameters at various locations in the WT drivetrain. Using these simulated data, the methodology presented in this paper is intended to prioritize candidate sensors prior to real-world testing in order to (1) reduce the number of sensors required and (2) enhance the utility of the subsequent instrumentation and real-world testing phases, which were not repeatable in this project due to resource constraints.

In order to generate the time-series dataset of the 6-DOF GB input loads (hereafter referred to as “target variables”) and the resulting deformations, misalignments, and rotational speeds of drivetrain components (hereafter referred to as “predictor variables”) to be analyzed in this investigation, Azzam et al., assembled models of the various WT components in a virtual MBS environment [9]. The rotor blades, as well as the tower, were modally decomposed after an initial modelling in finite elements (FE) [21,22]. In addition, various drivetrain components, e.g., main frame and gearbox planet carrier, which contribute to the predictor variables, were modeled as flexible bodies in the MBS environment, whereas micro-level flaws, such as material imperfections, were not incorporated in the simulations [9]. In order to subject the WT model to simulated wind fields, TurbSim, together with the AERODYN force element and a SIMULINK PI controller, was used as part of a co-simulation with MATLAB [23,24]. In their paper on the topic, Azzam et al. provide a more in-depth explanation of the simulation model, as well as a discussion of its validity with respect to the task of virtually sensing WT transmission input loads [9].

With the help of machine learning techniques, simulated data provide a valuable opportunity to gain insights into the influence of WT transmission input loads on deformations, misalignments, and other phenomena in the various components of the WT drivetrain that can later be measured using candidate sensors. Real measurements are expected to diverge from the simulated time-series data due to the presence of noise and various imperfections in the real-world test setup that cannot be fully replicated in a simulation environment. Therefore, it is important to emphasize the intended scope of use of the presented methodology as a sensor screening tool in the data-scarce design phase of a sensor set to be instrumented for subsequent real-world testing. Despite expected differences between real measurements and simulated data, we expect the general correlations present in measured data to agree with the correlations present in the simulated data. This is not only based on the simulation methodology presented by Azzam et al. [9] but also on the findings of similar investigations involving WT MBS models subjected to simulated wind fields, which demonstrated high correlation between simulated and measured data of WT transmission loads and displacements [25,26]. The current investigation is focused on prioritizing candidate sensors based on the influence of the transmission input loads on the respective phenomena they are intended to measure. Therefore, our aim was to gain insights into the relationships between said loads and phenomena in order to select sensors that are likely to capture highly pronounced, practically measurable effects of the transmission input loads where they manifest in the drivetrain.

In order to capture such relationships, machine learning techniques based on the prolific random forest (RF) ensemble method were utilized. Ensemble methods aim to aggregate the results of several models into a combined model, the performance of which exceeds that of any of its constituent models [27,28,29]. Machine learning using ensemble learning, or ensemble learning, has been successfully applied in numerous investigations to solve both classification and regression problems [27,30]. Dietterich attributed the success of ensemble methods to their statistical, computational, and representational advantages [31]. Several theoretical studies also provided insights into the performance drivers of ensemble learning, such as stochastic discrimination [32], strength correlation [33], margin theory [34], and bias–variance–covariance decomposition [35]. Fawagreh et al. identified three widely used ensemble approaches: boosting, bagging, and stacking [36]. Due to its robustness against model overfitting and noise in the data, bagging was identified as the approach of choice for this investigation [36,37]. Overfitting occurs when a model performs significantly better on training data compared to test or new data [38]. Bagging describes bootstrap aggregating [39], which consists of two main steps. (1) Bootstrapping involves training each constituent model on a randomly drawn sample of observations from the available data with replacement. (2) Aggregating involves an averaging of the outcomes of the constituting models to reach the output of the ensemble model. Combining bagging and the random selection of features [40,41,42], Breiman introduced the RF method, which consists of multiple decision trees grown not only using bootstrapped samples but also with a random sample of features to grow the decision trees [33]. Additionally, Breiman utilized the classification and regression trees (CART) technique [43] for variable selection at each node of the RF trees in order to introduce more randomness in the construction of the trees [33]. Loh provided the context for both classification and regression trees in his paper on the history of these algorithms [44]. Since its conception by Breiman [33], the RF algorithm has been one of the most commonly used ensemble methods in various investigations in the field of wind energy, as well as many other fields [45,46,47,48,49]. Due to its aforementioned advantages, particularly its speed, relative insensitivity to overfitting, and its variable importance measurement, the RF algorithm was utilized in this investigation to gain insights into the importance of candidate sensors or predictors.

In this paper, RF models were first developed to capture the complex relationships between the target and predictor variables during simulated operation. After training and testing the RF models on the task of estimating the target variables using all predictor variables, further tests were performed on the models with the aim of identifying the most important predictor variables for model performance. The simulations performed by Azzam et al. generated the dataset analyzed in this investigation [9]. The dataset contains the target and predictor variables as generated from a simulated operation of a Vestas V52 WT MBS model undergoing time-series wind field simulations [9]. With this paper, we aim to present a methodology for reducing the number of needed sensors, or predictor variables, for a 6-DOF WT virtual transmission load sensor system prior to real-world testing. Because real-world testing is costly to set up and repeat, we aimed to enhance the utility of subsequent real-world tests by providing a tool for prioritizing sensors to be installed, the signals of which contain the most information on the WT transmission input loads. Because real measurements are needed for many virtual sensing investigations where experimental data might be scarce or unattainable at early design stages, we also aimed to support the efforts of a large audience in the field with the outcomes of this paper.

The rest of this paper is organized as follows. In Section 1, we introduce and motivate the methods used in this investigation. In Section 2.1, we present the simulated data to be analyzed. In Section 2.2, we provide an overview of the proposed methodology, followed by an explanation of the data preprocessing steps in Section 2.3 and an explanation of the error estimation approaches used to assess the performance of the developed models in Section 2.4. In Section 2.5, we elaborate on considerations and challenges in practical implementation of the proposed method. In Section 3, we present the results of the investigation, followed by a discussion of the results in Section 4. The conclusions are then outlined in Section 5.

## 2. Data and Methodology

### 2.1. Data Description

**Figure 1:**
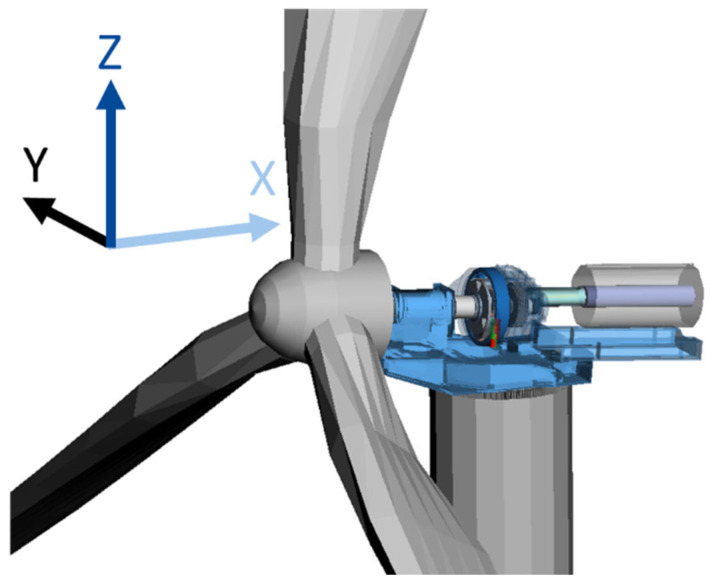
Illustration of the orientation of the x, y, and z axes of measurement with respect to the WT drivetrain.

The available dataset consists of 28 variables sampled at 200 Hz for a total duration of 60 min. Table 1 lists the available variables, indicating the subject (e.g., GB torque arms, wind, or generator), nature (e.g., displacement, misalignment, or rotational speed), location, and axis of the measurement, as well as whether each variable belongs to the target or predictor variable group. All measurements are with respect to the machine carrier of the WT. Furthermore, directional location data in Table 1 (where specified), such as right or left torque arms, are from the point of view of an observer facing the rotor from the position of the generator. Hereafter, the variable ID in Table 1 will be used to refer to the respective variable. Figure 1 indicates the orientations of the x, y, and z axes of measurement with respect to the WT drivetrain during the simulation. 

The dataset covers six wind field simulations of the Vestas V52 WT model, each resulting in 10 min of simulated data. Three nominal wind speeds corresponding to the cut-in, rated, and cut-out wind speeds (3, 12, and 25 m/s, respectively) were used to generate two of the six wind fields, with a different random seed for their respective turbulence models [9]. The three blade pitch angles in the current dataset are identical. Therefore, only one blade pitch angle was included in the analysis. For a more detailed explanation of how the dataset was generated, the reader is referred to the following publication [9].

### 2.2. Methodology Overview

**Figure 2:**
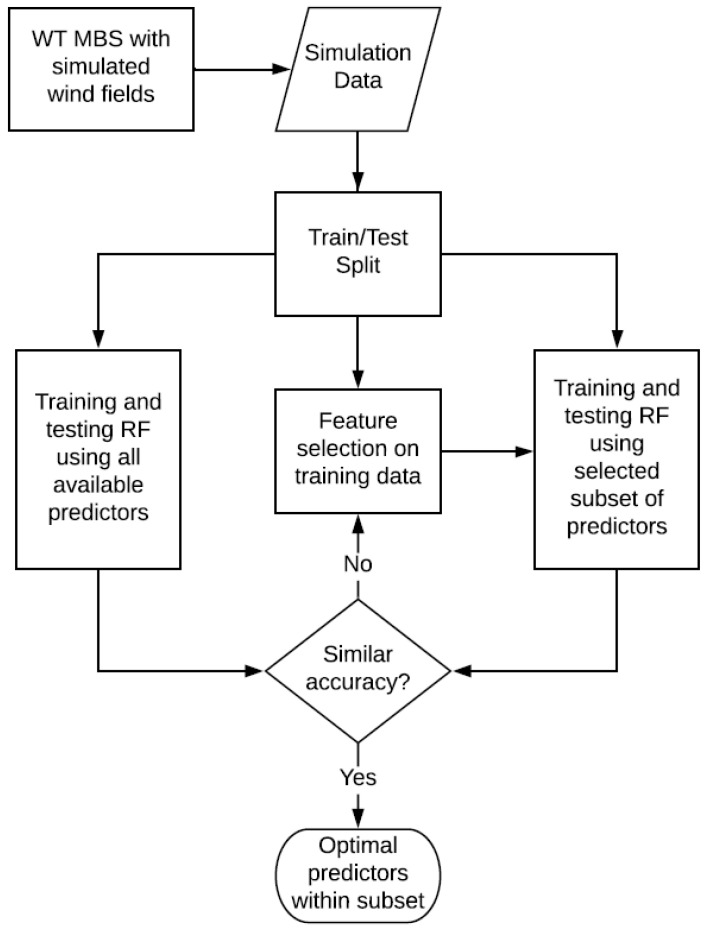
Overview of methodology.

The methodology for identifying highly influential predictor variables with respect to virtual sensing performance is based on an iterative training and testing of RF models in order to discover the patterns in the available data. Figure 2 provides an overview of the methodology. As mentioned earlier, in order to obtain the available data listed in Table 1, a model of the Vestas V52 WT was subjected to a simulated wind field in a virtual multibody simulation (MBS) environment [9]. The simulated wind fields were generated in accordance with design load cases from the IEC 61400 standard [50]. During simulated operation, time-series data of the 6-degree of freedom (6-DOF) transmission input loads were extracted, as well as signals from potential sensor locations covering deformations, misalignments, and rotational speeds at various drivetrain locations. These locations were selected based on an initial hypothesis of sensor locations suspected to be useful and practical for the envisaged transmission input load virtual sensor. However, as the computational cost of adding sensors to the WT model in the simulation environment is relatively low, additional locations could be investigated.

As shown in Figure 2, the process used to identify an optimal subset of sensor locations involves, first, the development of RF models that are trained and tested to estimate the desired target variables using all available predictor variables. Secondly, the methodology involves performing a feature importance analysis on the data in order to measure the influence of the respective predictor variables on the RF model accuracy relative to one another. At this stage, a prioritization of the available predictor variables can be achieved based on their influence or importance, and a subset containing highly important predictors can be selected. By training and testing RF models using only that subset of predictors and comparing the resulting performance to that of the RF models that had access to all available predictors in their development phase, a decision on whether to enlarge the chosen subset to contain more of the available predictors can be made. This decision would largely depend on the cost–accuracy tradeoff deemed appropriate by the development team. In Section 2.2.1, we provide a brief explanation of the RF algorithm [33], whereas in Section 2.2.2, we describe the feature importance analysis used in this investigation: the Boruta algorithm [51].

#### 2.2.1. Random Forests

In this investigation, RF regression models [33] were developed in order to capture the relationships between the predictor and target variable in the available data. The goal was to estimate the 6-DOF transmission input loads using the simulated predictor sensor signals listed in Table 1 as input. The available data introduced in Section 2.1 were used to train and test the RF models.

The RF model [33] consists of an ensemble of regression decision trees that, collectively, are able to learn to approximate a function. In this investigation, six such functions were desired, each capable of producing an accurate estimate of one of the 6-DOF transmission input loads using the predictor variables as input variables.

**Figure 3:**
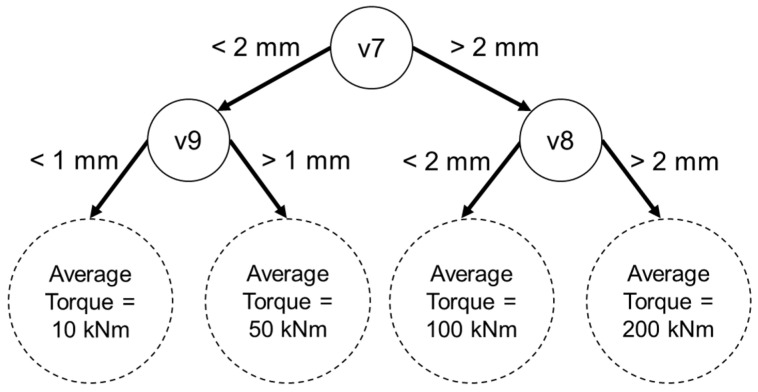
Example regression decision tree.

Figure 3 illustrates an example regression decision tree aimed at estimating torque based on v7, v8, and v9 as input variables. As a reminder, Table 1 lists the parameter measured by v7 to be displacement of the right hand-side GB torque arm along the x-axis, and Figure 1 demonstrates the orientation of the x-axis relative to the WT drivetrain. Starting from the root of the tree at the top of Figure 3, a given data point follows either the right or left path depending on its value for v7. It then follows the appropriate path depending on its value for v8 or v9 to the so-called leaves of the decision tree, illustrated as dotted circles in Figure 3. After a number of data points make their way from the root of the tree to one of its four leaves depending on the values of their respective input variables, the average of the target variable (in this example, torque) of the group of data points that reached each leaf is shown in the figure. This process is referred to as training the decision tree. In the example tree shown in Figure 3, the average torque of the data points that reached the leftmost leaf is 10 kNm. If a new data point with an unknown torque reaches the leftmost leaf; then, the decision tree estimates that the torque for that data point is 10 kNm. A regression RF model contains several such regression decision trees with different numbers of branches and different splitting thresholds at each branch to, collectively, reach a more accurate estimation of the target variable as compared to the estimation reached by an individual decision tree. Breiman [33] provides more information about random forests.

In order to develop an RF model, the investigator must design its architecture by deciding on characteristics of the model, such as the number of decision trees to use, as well as other so-called hyperparameters that influence the performance of the model and the computational effort it will require during its lifecycle. In this investigation, multiple iterations of trial and testing led to a more favorable set of hyperparameters in a process called hyperparameter tuning. Experience and domain knowledge guide the process of hyperparameter tuning by helping to prioritize certain model hyperparameters. For example, two of the most influential RF hyperparameters are sample size and number of tried features at each decision tree split (hereafter referred to as “mtry” [52]. Sample size refers to the number of data points selected from the entire set of training data to be used for training each decision tree in the random forest. The parameter “mtry” refers to the number of candidate predictor variables assessed for each split in the decision tree or, in other words, each point in a given tree where a data point follows the right or left path, as shown in Figure 3, during the training phase of an RF model. Whereas decreasing either of these hyperparameters is likely to have a negative impact on decision tree accuracy and, in turn, the RF model accuracy, increasing them may result in model overfitting [52]. Overfitting describes a model that reaches significantly higher accuracies on data points used for training than other data points, for example, those used for testing the model. For some hyperparameters, there is not a clear consensus on the degree to which they influence model accuracy. For example, the number of decision trees in a random forest is one such hyperparameter that has been set to a computationally feasible large number in previous investigations [52,53,54]. In their paper on the topic, Probst et al. explain some best practices for RF hyperparameter tuning, as well as the potential effects of tuning the different hyperparameters on RF model performance [52].

#### 2.2.2. Identification of Influential Predictor Variables Using the Boruta Algorithm

Whereas the RF models aimed to estimate the 6-DOF transmission input loads, in this section, we present the process by which the predictor variables with the biggest impact on model accuracy are identified. This task was achieved by following the Boruta algorithm [51]. A copy of each predictor variable was randomly shuffled, resulting in 18 distorted versions (so-called shadows) of the predictor variables. The shadows were then added to the set of original predictor variables, and a statistical test [51] was used to iteratively assess whether an original predictor variable will prove to be less important to target variable estimation than the random shadows. A given predictor variable is deemed unimportant if, on average over several iterations, it is found to be less important than the most important shadow variable. For the variables that are deemed important, the result of the analysis can also be used to identify highly important predictor variables that are most influential with respect to the estimation accuracy. Kursa and Rudnicki provide more details on the Boruta algorithm and the calculation of the importance value [51].

### 2.3. Data Preprocessing

Because the predictor variables in the available dataset vary in their scale of measurement, certain data preprocessing techniques could enhance the performance of the feature importance methodology. In their often-cited paper on the topic of bias in RF variable importance measures, Strobl et al. demonstrated that the existence of predictor variables with significantly varying scales of measurement can be a source of bias when identifying important predictors [55]. As a countermeasure, the predictor data used in this investigation were standardized. Standardization was performed on the data belonging to each predictor variable using the following formula:

(1) $$Z=\frac{X_{\left(i\right)}-u}{\sigma }$$

where *X*, *u*, and *σ* refer to the sample of each predictor variable, its mean, and standard deviation, respectively. The available data were then split into 2 datasets: training and testing. The training set contained 50% of the data, whereas the testing set contained the remaining 50%. This step was performed in order to assess and monitor model overfitting if it takes place. As described in the Introduction, model overfitting takes place when the model performs significantly better on data used in training as compared to new data. Whereas the training set is used to train the models, the testing set would not be used in the training phase. This is not only to use the testing set to assess the performance of the model but also to ensure that overfitting has not taken place. Because, in the case of the testing set, performance is assessed on new data from the point of the view of the trained model, a comparison to the model performance using the training set would indicate model overfitting and, in turn, indicate the need for a revision of the model training procedure.

In addition, the aforementioned scaling was performed in such a way as to avoid data leakage or leakage of information from the testing set to the training set. The mean and standard deviations used to scale both the training and testing sets were obtained only from the training set. This prevented such information from the test set from influencing the training set and potentially causing model overfitting.

### 2.4. Error Estimation

In order to assess the performance of the proposed methodology, the error of the developed models with all predictors and with only the highly important subset of predictors was estimated using two approaches: the coefficient of determination, or R^2^ score, [56] as well as the LRE score [9]. First, the R^2^ score was calculated for each of the 6-DOF loads by applying Formula (2) to the target values from the test dataset and model estimations [56]:

(2) $$R^{2}\left(y,\hat{y}\right)=1-\frac{\sum_{i=1}^{n}{\left(y_{i}-{\hat{y}}_{i}\right)}^{2}}{\sum_{i=1}^{n}{\left(y_{i}-\bar{y}\right)}^{2}}$$

where $\;y_{i}$, ${\hat{y}}_{i}$, *n*, and $\bar{y}$ represent the *i*th expected value, the *i*th estimated value, the quantity, and the mean of the expected values of the target variable, i.e., $\bar{y}=\frac{1}{n}\sum_{i=1}^{n}y_{i}$, respectively.

In addition, an approach recently developed by Azzam et al. [9] was utilized to estimate the error of the estimated loads by the RF models used in this publication with all predictors and with only the highly important subset of predictors. Following this approach, statistics covering the minimum, maximum, and average of each of the 6-DOF transmission input loads, listed in Table 2, were extracted from the simulated data at nominal wind speed. For example, the forces along the y-axis typically fluctuate around an average value close to zero [9]. Azzam et al. argue that if mean absolute error [57], for example, is calculated and compared to the average values of the respective loads, this would result in an inflated estimation of error for a load such as Fy. Alternatively, comparing the error to the maximum value of the respective load would result in an underestimation of error. By scaling the mean absolute error against the median of the three statistics (minimum, maximum, and mean), an error relative to a simple statistic representing the loads at nominal wind speed can be calculated, taking into account the problems mentioned above with comparing the error to only one statistic. Therefore, in addition to the commonly used R^2^ score, the load relative error (LRE) was used in this investigation to assess the performance of the developed models, whereby the LRE of the *i*th estimated value for each target variable was calculated using Formula (3) [9]:

(3) $${\mathrm{LRE}}_{i}=\frac{\;y_{i}-{\hat{y}}_{i}}{\mathrm{median}\left(\left|y_{max}\right|,\left|y_{min}\right|,\left|\bar{y}\right|\right)}$$

where $y_{max}$ and $y_{min}$ are the maximum and minimum values of the target variable, respectively.

The simulated load statistics selected for the calculation of the LRE for each transmission input load are underlined in Table 2 to facilitate interpretation.

### 2.5. Considerations and Challenges in Practical Implementation

In this investigation, simulation data were used to screen candidate sensors prior to real-world testing following the methodology outlined in Figure 2. In order to assess the performance of the proposed method, RF models were trained and tested on the task of virtually sensing the 6-DOF transmission input loads before and after the application of the proposed method to identify a relatively important subset of simulated predictor variables. Therefore, the accuracy of the RF models is a function of the validity of the simulation model and its resulting data. Because the unconditional validity of a simulation models is refuted by most experts [58,59,60,61], the validity of a simulation model can only be defined within the bounds of a project and its intended application [9,62]. As mentioned earlier, the presented methodology is intended as a sensor screening tool in the data-scarce design phase of sensor sets to be instrumented for subsequent real-world testing to develop a virtual 6-DOF WT transmission input load sensor. Real measurements are expected to diverge from the respective simulated time-series data due to the presence of noise and various imperfections in the real-world test setup that cannot be fully replicated in a simulation environment. The performance of the RF virtual sensor models developed in this investigation provides insights into the possibility of learning the patterns between the predictor and target variables of the envisaged virtual sensor system using RF regression models. However, real-world measurements are still needed to assess whether this ability applies to real-world data. Therefore, the next step in this project was to instrument and test two drivetrain configurations of the Vestas V52 as part of a measurement campaign aimed at further developing the envisaged 6-DOF transmission input load virtual sensor. For that purpose, the proposed method uses simulated data to provide a valuable opportunity to screen different candidate sensors of the envisaged virtual sensor system prior to the resource-intensive and less flexible real-world test. In the remainder of this subsection, we identify some considerations for the practical implementation of this method.

Although feature scaling can have a considerable impact on the performance of certain ML algorithms, e.g., artificial neural networks (ANN) and support vector machines (SVM), it can have less of an impact on other algorithms [55,63,64]. Through experiments comparing the performance of ML algorithms on different datasets with and without data preprocessing, Misra et al. found random forests to be one of the top-performing algorithms without feature scaling [63]. Experiments by Ahsan et al. also showed the performance of RF to be relatively insensitive to feature scaling as compared to distance-based algorithms, such as SVM, and algorithms relying on stochastic gradient descent, such as ANN [64]. This is a logical finding, given that algorithms that rely on stochastic gradient descent, such as ANNs, use the same learning rate for every parameter [65]. On the other hand, the tree-based algorithm used in RF makes each splitting decision within a given tree based on only one variable at each node of the tree [43]. Therefore, the performance of RF models tends to be relatively insensitive to feature scaling [64,66]. However, the effect of data scaling may vary depending on the intended use of a given algorithm. A relevant example is the investigation by Strobl et al. [55], where experiments showed that using the RF algorithm described by Breiman [33] in variable importance investigations may lead to biased findings when predictor variables vary in their scale of measurement. Therefore, to mitigate against bias in the variable importance outcomes of this investigation, data scaling was used to limit variations in the scale of measurement of the predictor variables. The two most widely used data scaling algorithms are the min–max algorithm and the z-score algorithm, also known as the standard scaler algorithm [67]. Whereas the effect of scaling may vary with different ML models, investigations on the effect of different scaling methods on the performance of ML models aim to identify suitable scaling algorithms for the most widely used ML models [64]. For example, in their investigation of the influence of different methods of scaling on heart failure patient datasets, Balabaeva et al. achieved a higher RF performance using the standard scaler method as compared to several other methods, including the min–max algorithm [67]. In this investigation, standard scaler was used to scale the available data. 

As z-score scaling is sensitive to outliers, it is important to assess the presence of outliers in the data before scaling [68]. In the case of outliers, several approaches can ensure robustness of the scaling operation, such as robust data scaling, where outliers are excluded from the calculation of the mean and standard deviation used in Equation (1) to scale each variable. Because outliers are data points not conforming to an expected behavior, an understanding of the expected behavior is needed to discern outliers. Whereas domain expertise can be used to detect outliers in the data, the process of identifying potential outliers can be automated to assist in investigations involving high-dimensional big data [69]. Thudumu et al. provide a comprehensive survey of anomaly detection techniques for high-dimensional big data applications [69]. Due to the relatively small amount of simulated data analyzed in this investigation, as well as the domain knowledge of the authors, an exploratory data analysis involving visualizations of the data was sufficient to rule out the presence of outliers. For applications involving large amounts of data, we recommend the use of automated outlier detection techniques prior to the application of the proposed method.

## 3. Results

Six RF regression models were trained to estimate the respective 6-DOF transmission input loads, taking the predictor variables as input. The available data from six simulated wind fields, each lasting 600 s, were used to train and test the RF models. The RF models demonstrated varying levels of skill in estimating the target 6-DOF loads, reaching R^2^ scores [56] for moments about the x-, y-, and z-axes of 0.99, 0.95, and 0.99, respectively, and R^2^ scores for forces along the x-, y-, and z-axes of 0.81, 0.75, and 0.82, respectively.

**Figure 4:**
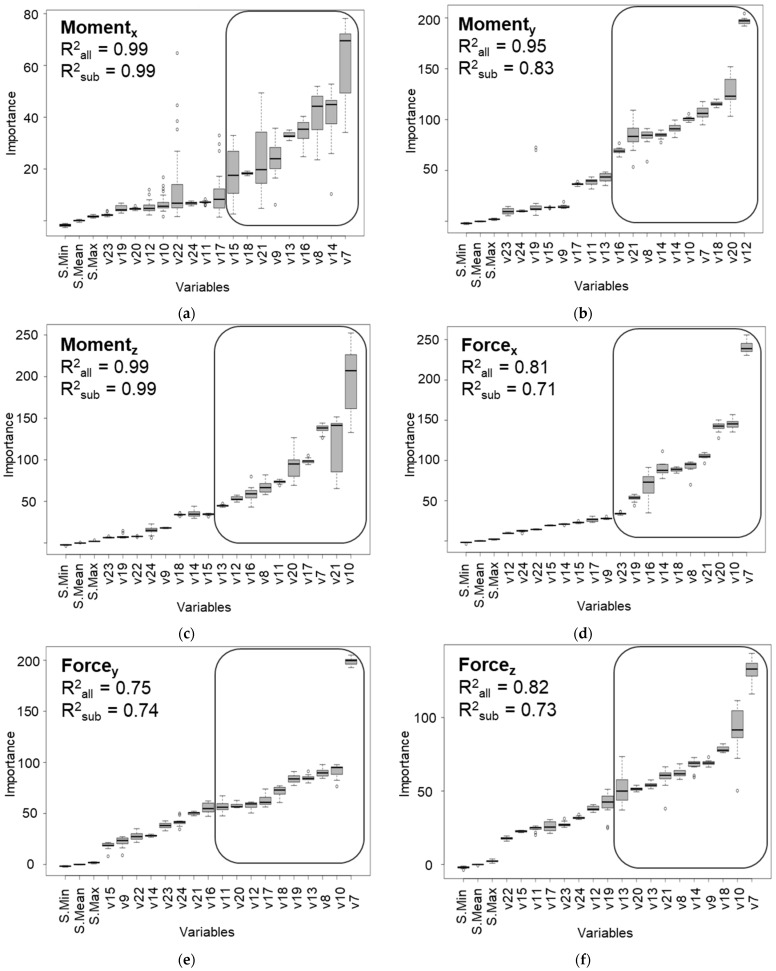
Importance of predictor variables using random forests and the Boruta algorithm [51]. The panels show the variable importance results for each of the target variables listed in Table 1 (**a**–**f**), covering variables v1–v6, respectively.

In order to identify the most influential predictor variables with respect to estimation accuracy, the Boruta algorithm [51] was used to iteratively remove predictors less important than the shadows in 6-DOF load estimation. This process was applied for each of the 6-DOF transmission input loads. The results indicate that for each RF model estimating a specific target variable, a subset of available predictors is of particularly high importance with respect to model accuracy. The Boruta algorithm was applied once for each of the six target variables, resulting in the six plots shown in Figure 4. Also shown in the figure, the minimum, mean, and maximum importance values of the shadows are indicated as S.Min, S.Mean, and S.Max, respectively. In Figure 4, it is clear that all predictors were found to be important, as none have an importance lower than S.Max. In other words, no shadow variable was found to be more important than any of the available predictor variables. However, the plot shows that a subset of the available predictor variables (shown encompassed within a rounded rectangle) was found to be highly important in comparison to the remaining predictors for estimating each of the 6-DOF transmission input loads. 

In order to verify that these subsets indicated the optimal sensor locations, RF models were trained using only the predictor variables within the subsets. The resulting R^2^ scores for moments about the x-, y-, and z-axes were 0.99, 0.83, and 0.99, respectively, and those for forces along the x-, y-, and z-axes were 0.71, 0.74, and 0.73, respectively.

**Figure 5:**
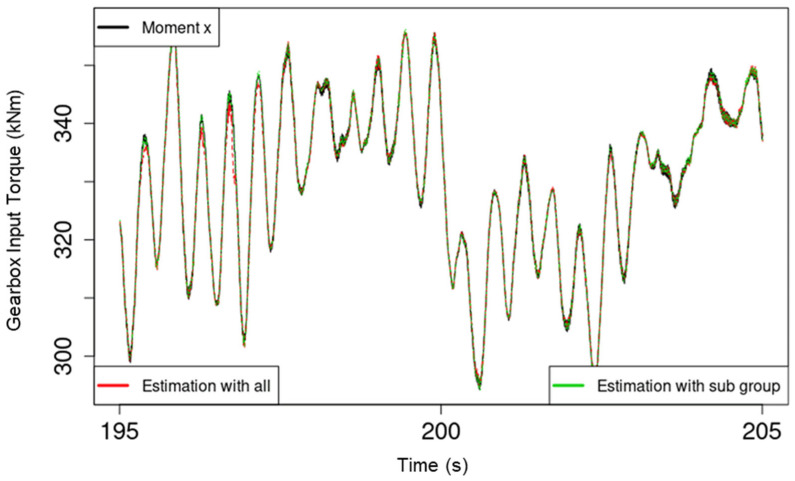
Plot of torque, model estimations with all predictors, and with only the highly important subset of predictors.

**Figure 6:**
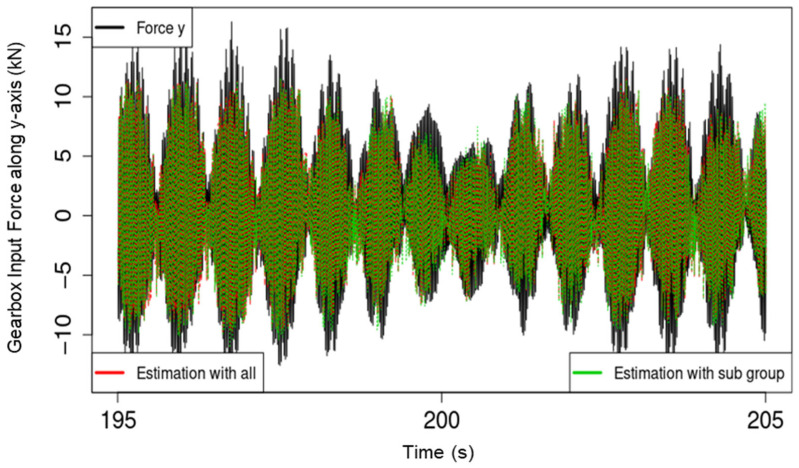
Plot of force along the y-axis, model estimations with all predictors, and with only the highly important subset of predictors.

For comparison between the performance of the RF models using all available predictor variables and the models that only used the subset of highly important variables, two R^2^ scores, R^2^_all_ and R^2^_sub_, are provided in the plots in Figure 4. The specific target variable of the respective plot in Figure 4 is also indicated in the top left corner within the plot for more clarity. Whereas the difference between R^2^_sub_ and R^2^_all_ varied for the different target variables, R^2^_sub_ remained within 13% of R^2^_all_ despite using between 50% and 55% of the available predictors to train and test the RF models responsible for R^2^_sub_. To further demonstrate the performance of the developed models, Figure 5 and Figure 6 show plots of the loads, model estimates using all predictor variables, and estimates using only the respective important subset for torque and force along the y-axis, respectively. The figures show 10 s of data from the testing set.

Figure 4 also shows that some predictor variables are important for the estimation of all 6-DOF transmission input loads. In particular, torque arm displacement along the x-axis is a highly, if not the most, important predictor for all target variables.

**Figure 7:**
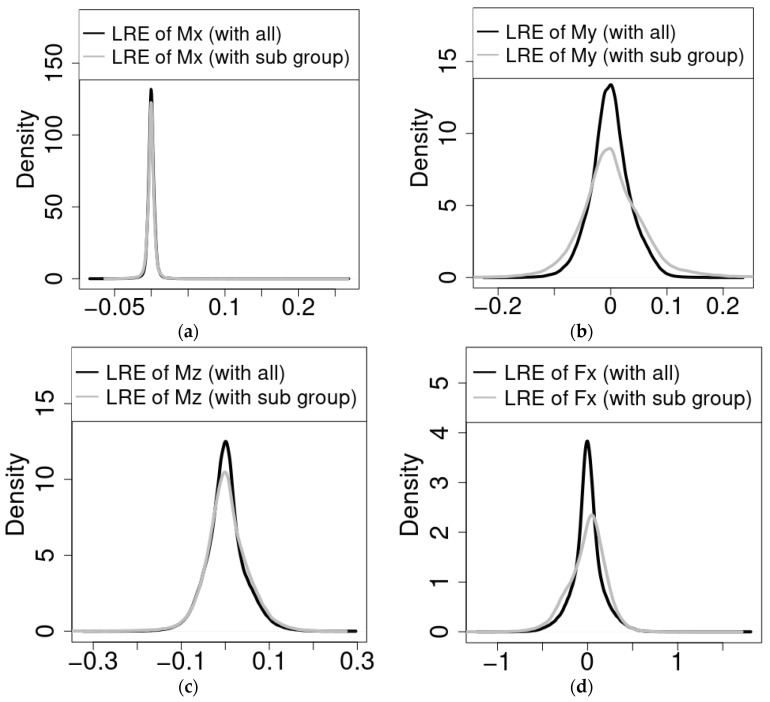

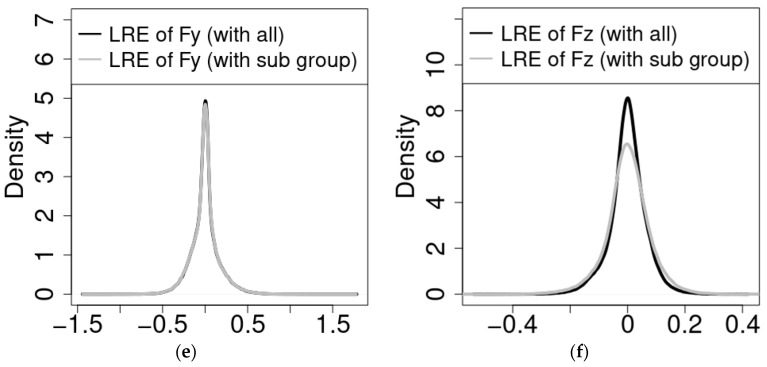
Load relative error [9] distributions for estimated transmission input moments with all predictors (black) and with only the highly important subset of predictors (grey). The panels show the LRE for each of the respective target variables listed in Table 1 (**a**–**f**), covering variables v1–v6, respectively.

In order to further demonstrate and assess the performance of the proposed methodology on the entirety of the test dataset, the LRE of the estimated values for each target variable using all predictors, as well as using only the highly important subset of predictors, was calculated. The distributions of the respective calculated LREs for each of the six target variables are shown in Figure 7.

## 4. Discussion

The results indicate a considerable capability of the RF models in virtually sensing transmission input moments around the x-, y-, and z-axes: v1, v2, and v3. This is demonstrated both by the high achieved R^2^ scores of the RF models that used all available predictor variables, as well as those models that used only the respective highly important subsets for estimating the input moments. Although the model for moments around the y-axis suffered almost a 13% reduction in R^2^_sub_ score as compared to the R^2^_all_ score, its R^2^_all_ score was a high 0.95, indicating the capability of the developed model to capture the relationships between the available predictor variables and this target variable. The fact that the presented methodology achieved a 13% reduction in performance with a 45% reduction in used predictor variables also indicates its capability of identifying and prioritizing important predictors.

The relatively lower sensitivity of the developed RF models to input forces (force along the x-, y-, and z-axes) is likely be due to the drivetrain configuration of the Vestas V52 WT MBS model, which was used to generate the data. The WT drivetrain has a four-point bearing suspension configuration, which is designed to eliminate all GB input loads, apart from torque, using the main bearing configuration of the drivetrain. This would also explain the contrasting performance between the model estimates of torque and those of the force along the y-axis, as shown in Figure 5 and Figure 6, respectively. The availability of more data for training covering more test scenarios through a measurement campaign that is also planned as part of this project is likely to further improve model performance and generalizability. Overall, the results show high promise for the use of regression RF models in virtually sensing transmission input moments and especially torque.

The results of the feature importance analysis also demonstrate the promise of the presented methodology, shown in Figure 2, for the prioritization and reduction in the needed number of sensors or sensor locations for the envisaged 6-DOF GB virtual load sensor. Once again, the performance with respect to torque stands out, as the reduction in the predictor variables available to the RF model resulted in a negligible accuracy difference. Whereas the RF models of the remaining loads suffered in terms of achieved accuracy after reducing the number of available predictor variables, the differences were within 13% of the accuracies of the models with access to all predictors, as shown in Figure 4 and Figure 7. The results are already useful in designing the envisaged virtual sensor setup of the planned measurement campaign, as they enable the project team to prioritize the sensors to be installed prior to the campaign. For example, torque arm displacement along the x-axis is currently of high priority as a result of the analyses presented in this paper. As changing the setup during the campaign is prohibitively costly due to the lack of accessibility of certain locations and the loss of valuable testing time, the method proposed in this paper will be used to optimize the use of the available resources for the setup of the measurement campaign.

## 5. Conclusions

In this paper, we presented a methodology for screening candidate sensors at different locations of the drivetrain for virtually sensing WT gearbox input loads prior to real-world testing. This was achieved with the use of RF models trained and tested on simulated data from a multibody simulation of a WT model. The main conclusions are as follows:

1. The results demonstrate the possibility of learning patterns between the predictor and target variables of the envisaged virtual sensor system using RF regression models and simulated WT operational data prior to real-world tests.
2. The developed RF models demonstrated a higher accuracy in virtually sensing WT transmission input moments as compared to transmission input forces. The performance of the RF model developed for virtually sensing torque resulted in the highest R^2^ score. Performance may vary when using real measurement data to train and test the models due to the presence of noise and other deviations. However, the results obtained using simulated data provide insights into the challenges ahead in virtually sensing the 6-DOF transmission input loads, given the design of the drivetrain under investigation.
3. The presented methodology is able to screen candidate sensors in the data-scarce design phase of sensor sets to be instrumented for subsequent real-world testing for virtually sensing 6-DOF transmission input loads.

We aim to utilize the methodology presented in this paper to identify high-potential sensor locations and types of an envisaged virtual sensor system for estimating WT 6-DOF transmission input loads.

## Figures and Tables

**Table 1:** Available variables and their respective attributes.

ID	Subject(s)	Measurement	Axis of Measurement	Predictor/Target
v1	GB input load	Moment	x	T
v2			y	
v3			z	
v4	GB input load	Force	x	T
v5			y	
v6			z	
v7	GB torque arm (right)	Displacement	x	P
v8			y	
v9			z	
v10	GB torque arm (right)	Angular misalignment	x	P
v11			y	
v12			z	
v13	GB torque arm (left)	Displacement	x	P
v14			y	
v15			z	
v16	GB torque arm (left)	Angular misalignment	x	P
v17			y	
v18			z	
v19	Generator mount (left)	Displacement	z	P
v20	Generator mount (right)			
v21	Wind	Direction	Nacelle axis of rotation	P
v22		speed	x	
v23	Generator	Rotational speed	x	P
v24	Blade ^1^	Pitch angle	Blade axis of rotation	P

^1^ Only one pitch angle is included because the three blade pitch angles are identical in the dataset.

**Table 2:** Simulated load statistics at rated wind speed.

Simulated Load Statistics at Rated Wind Speed (Units: N, Nm)		
Min.	Max.	Avg.
−7898	1050	−3222
−17,972	15,556	−81
−53,850	−26,380	−41,074
−380,056	−41,669	−257,847
22,881	32,429	27,368
4555	45,827	30,124

## Data Availability

The data are not publicly available.
